# Tuberculosis and the War

**Published:** 1919-07-12

**Authors:** 


### TUBERCULOSIS AND THE WAR.

### Some Views on Future Developments.

THE wide question of tuberculosis under after-war conditions and the measures to be taken with regard to the ex-Service man were discussed at length on Saturday, June 28, at the final meeting of the Conference in relation to public health.

Considerable criticism was directed against the present-day sanatorium, which is described as inefficient, unsatisfactory, and lacking in uniformity of treatment. The accommodation is insufficient and bad, and soldiers and sailors suffering from tuberculosis in many cases refuse to go to them in spite of all the urging of the Government. Sanatoria are more like prisons than anything else—and useless prisons, too—for the treatment is too short to have any permanent effect.

### ESSENTIALS IN SANATORIA.

Far too much attention is paid to what is in the patient's chest and far too little to his psychology, said one speaker, and another cited the case of a sanatorium in which the only amusements were a cracked and worn-out set of bowls and a gramophone. A sanatorium to be successful must, first, be competently run by a resident medical officer; and, secondly, must do everything in its power to keep the patient's mind occupied and cheerful. A suggested reason for the objection of ex-Service men to these institutions is that a bad impression has been created in the public mind by the number of deaths which have there taken place—a number swollen by the late period at which patients are usually admitted.

The importance of useful employment for patients was emphasised by several speakers. It is quite possible for sufferers in all but the most advanced stages to earn a livelihood by pursuing some sort of trade, and it was suggested incidentally that there is evidence to prove that industry forms a more suitable occupation than land work. Whatever work is taken up must, however, be of a really useful and not merely decorative character, so as to satisfy the common sense of the person following it. But a difficulty arises in that labour demands in sanatoria are limited, and attempts that have been made to create employment have met with small measure of success. It is not fair, also, that partially disabled workers should have to compete with sound workers in the market.

### SEGREGATION OF PATIENTS.

Segregation of tuberculosis patients was agreed to be both necessary and effective in restricting the spread of the disease, but the present form of segregation in which all forms of family life are rendered impossible is rather called in question, and attributed as another reason for the ex-Service man's objection to sanatorium treatment. At this time, when housing schemes are being put forward throughout the whole country, it was suggested that a certain number of houses in each town shall be specially constructed for the reception of tuberculosis sufferers and their families. If this is impossible owing to the obstruction of timid committees, the Red Cross or other Society might start a national scheme for dealing with the families of patients, and a third suggestion was to the effect that adequate separation allowances—such as have been paid to men on service—should be paid to those wage-earners who are admitted into sanatoria, the advantages of this scheme being earlier admission and chances of more prolonged treatment.

Dr. S. Vere Pearson struck a bold note by declaring that the nationalisation of the land is the only way of eradicating tuberculosis. Nobody can enjoy a more spacious life, he said, until the evils of land monopoly are broken down. Eliminate poverty and the root causes of all ill-health will be done away with too. Institutions which are financed out of public funds and wrong methods of taxation defeats their own end by simply emphasising the wrongs suffered by society, which prevents it from following any of the ordinary rules of health.

It was urged, on the other hand, by Dr. Wall that after a period in a sanatorium insanitary conditions are of less importance than the obviation of undue physical strain. Fresh air figures too largely in modern treatment, because under it the medical man cloaks his ignorance of other measures.

Lt.-Col. Mullock Hart voiced the expression of a Conference he had attended in Paris that it is remarkable that tuberculosis is not more prevalent among Service men. This may possibly be accounted for by the fact that the number of actual cases tends to balance the number of those with whom the outdoor life killed the germs that are prevalent in almost everybody. In the course of his war service he made a careful analysis of the age at which tuberculosis is most prevalent. This is undoubtedly between twenty-one and thirty, from which he drew 55 per cent. of his patients. Next came thirty-one to forty years with 24 per cent. About 14 per cent. were between sixteen and twenty-one, and 6 per cent. between forty and fifty-seven.

### TUBERCULAR EX-SERVICE MEN.

Lt.-Col. Nathan Raw foreshadowed the scheme of the Government Commission now sitting in aid of tubercular ex-Service men. The Government is determined that the fullest pension shall be paid to such men, and when the scheme is put into operation it will be found that the latest scientific experience has been incorporated in it. Of the 35,000 to 40,000 men affected a large proportion are capable of doing useful work, and will be taught a trade which will arrest and cure the disease, or encouraged to start businesses for themselves. In those cases which are too advanced to resist, comfortable homes will be found in which the sufferers will be able to spend their last days. What is most needed at the present time is, first, concerted national action; and, secondly, abolition of the idea that tuberculosis is incurable—an idea which he described as "most dangerous to the country."

The backward education of the public was an undercurrent of complaint with nearly every speaker. The general practitioner is ignorant on this subject, and has no time in which to keep abreast of it, and a speaker told how on one occasion, when he had agreed to admit a patient to a sanatorium on the word of a country doctor that the case was in its early stages, the patient arrived dead in the cab.

### THE SITUATION IN IRELAND.

The Marchioness of Aberdeen outlined the situation in Ireland, and said that the educational campaign conducted there a few years ago was satisfactory, but since then matters have stood still, so that the workers now are filled with disappointment and anxiety. Very little provision is made for children, and scarcely any for advanced cases.

A plea for greater research in and more attention to non-pulmonary tuberculosis was made by Dr. Gauvain, whose sufferers he described as "the most neglected class in the country." He cited the case of a sailor who had contracted surgical tuberculosis as the result of a blow received while coaling ship. He was discharged from the Navy and granted a pension—7d. a day for one year. Now is the opportunity, urged Dr. Gauvain, for something effective to be done. The present policy of *laissez faire* means disaster, and one or two properly equipped institutions can deal with the whole question.

